# Educational outcomes in siblings of childhood leukemia survivors: Factors associated with school difficulties and comparison with general population

**DOI:** 10.1002/cam4.6821

**Published:** 2024-01-10

**Authors:** Cindy Faust, Pascal Auquier, Virginie Gandemer, Yves Bertrand, Marie‐Dominique Tabone, Sophie Ansoborlo, André Baruchel, Jacinthe Bonneau, Jean‐Hugues Dalle, Pascal Chastagner, Justyna Kanold, Maryline Poirée, Alexandre Theron, Laura Olivier, Isabelle Pellier, Gérard Michel, Julie Berbis

**Affiliations:** ^1^ UR 3279, CERESS – Health Service Research and Quality of Life Center Aix‐Marseille University Marseille France; ^2^ Department of Pediatric Hematology‐Oncology University Hospital of Rennes Rennes France; ^3^ Department of Pediatric Hematology‐Oncology University Hospital of Lyon Lyon France; ^4^ Department of Pediatric Hematology‐Oncology A. Trousseau Hospital, AP‐HP Paris France; ^5^ Department of Pediatric Hematology‐Oncology University Hospital of Bordeaux Bordeaux France; ^6^ Department of Pediatric Hematology‐Oncology, Robert Debré Hospital GHU AP‐HP Nord Université Paris Cité Paris France; ^7^ Department of Pediatric Hematology‐Oncology Children's Hospital of Brabois Vandoeuvre Les Nancy France; ^8^ Department of Pediatric Hematology‐Oncology CIC Inserm 501, University Hospital of Clermont‐Ferrand Clermont‐Ferrand France; ^9^ Department of Pediatric Hematology‐Oncology University Hospital L'Archet Nice France; ^10^ Department of Pediatric Hematology‐Oncology University Hospital of Montpellier Montpellier France; ^11^ Department of Pediatric Hematology‐Oncology University Hospital of Toulouse Toulouse France; ^12^ Department of Pediatric Hematology‐Oncology University Hospital of Angers Angers France; ^13^ Department of Pediatric Hematology‐Oncology Timone Children's Hospital and Aix‐Marseille University Marseille France

**Keywords:** epidemiology and prevention, pediatric cancer, psychosocial studies, survival

## Abstract

**Background:**

To investigate the educational outcomes of siblings of childhood leukemia survivors, explore determinants of school difficulties, and compare the rates of repeating grades between siblings and the general population.

**Methods:**

A cross‐sectional study of childhood leukemia survivors' siblings recruited through the Leucémies de l'Enfant et de l'Adolescent cohort, a French long‐term follow‐up program, was conducted, and education‐related data were obtained via self‐report questionnaires. Adjusted logistic regression models were used to identify variables associated with school difficulties and time since diagnosis. Rates of repeating a grade in middle school were compared between siblings and the general population of the same generation.

**Results:**

A total of 564 siblings with a mean time from diagnosis of 14.1 ± 6.4 years were included, among whom 139 (24.6%) repeated a grade, at an average of 6.4 ± 4.5 years after diagnosis. In multivariate analysis, the risk factors for repeating a grade were older siblings (odds ratio [OR] 2.3, *p* = 0.006), family financial difficulties (OR 2.8, *p* = 0.008), and history of repetition in survivors (OR, 2.5, *p* = 0.001). Sibling hematopoietic stem cell donors were at greater risk of repeating a grade long‐term after diagnosis (*p* = 0.018). Overall, siblings did not have a higher risk of educational delays at the end of middle school than the general population.

**Conclusion:**

Although the results are reassuring, socioeconomic and cancer‐related factors may have an impact on siblings' schooling long after diagnosis. Paying attention to siblings contributes to identifying the most vulnerable families, allowing more attention and appropriate resources to avoid long‐term repercussions. Additionally, supportive and targeted interventions can be developed to improve the organization of education and the health care system.

## 1 INTRODUCTION

A pediatric cancer diagnosis is a major life‐changing event that has unpredictable effects on all family members of the ill child, such as the parents and any siblings. In addition to hospitalization and prognosis uncertainty, the whole family faces substantial changes in their daily life, routines and familial roles, and responsibilities.^1^, ^2^ Research on siblings of childhood cancer survivors has steadily increased over the years, and several studies have documented the influence of this particularly stressful experience on their life, showing strong negative emotions (e.g., anger, guilt, and sadness), additional household responsibilities, and a decreased availability of their parents, resulting in a sense of parental absence.^3^, ^4^, ^5^ Although the literature focusing on the impact of these challenges on siblings' physical and psychosocial well‐being showed that most of them adjust well over time,^6^, ^7^ siblings may still be at risk of experiencing long‐term repercussions.^8^ In the matter of emotional and psychosocial problems, siblings can experience post‐traumatic stress symptoms^9^, ^10^ and disruptions in family functioning.^11^, ^12^ Regarding their quality of life (QoL), few studies have reported impairments in perceived QoL among siblings of childhood cancer survivors.^13^, ^14^, ^15^

However, concerning siblings' education and school performance, findings in the short term after diagnosis may be conflicting.^16^, ^17^ Indeed, several studies have reported that siblings were unable to concentrate during class and homework, possibly due to their fear about their brother or sister illness, resulting in decline and problems in academic achievement.^3^, ^18^, ^19^ Similarly, siblings' school attendance was found to be negatively affected, as they missed significantly more days of school than their peers.^20^ In those studies, parents often attributed siblings' educational difficulties to being less available to assist them with their homework and manage their school life, whereas frequent absenteeism may occur due to siblings' greater desire to be with and support the ill child. Moreover, poor school attendance has been shown to predict poor academic achievement, decreased probability of high school graduation, and reduced access to postsecondary education. However, while some of the siblings' needs seem to be neglected or underprioritized, other studies indicated that siblings of childhood cancer survivors continued to attend school on a regular basis; moreover, for some of them, their grades were unaffected.^21^

In the context of childhood acute leukemia (AL), which is the most frequent pediatric cancer diagnosis^22^ with a survival rate exceeding 80% on average,^23^ the population of patients surviving childhood leukemia continues to grow. Therefore, the implementation of a long‐term follow‐up is essential for not only better understanding long‐term effects among survivors, but also allowing research to maintain attention and to improve knowledge on the experiences of family members after facing pediatric AL within the family. Although the long‐term consequences of the disease on siblings' education have not yet been thoroughly explored, a previous study conducted on the impact of childhood AL on survivors' schooling long‐term after diagnosis reported that almost 30% had repeated a grade in the 4 years postdiagnosis, and while they were at greater risk compared to siblings, one of the risk factors for repeating a grade was the socioeconomic situation of the family at the time of diagnosis, an element that was unequivocally shared by the siblings.^24^ Therefore, even if siblings experienced the disease in a very different way compared to the survivors, it seems important to question to what extent siblings are impacted compared to the general population.

Overall, all those contextual elements support the relevance of our study to focus on healthy brothers and sisters and to explore their educational outcomes but also to assume that even if the ill child has survived, the experience of childhood AL, which is associated with different factors of vulnerability, can continue to have a negative impact on siblings' schooling in the long term.

Our general purpose was to analyze the schooling of siblings of pediatric AL survivors. The main objective was to describe siblings' school difficulties and to explore siblings', familial, and cancer‐related factors associated with postdiagnosis grade repetition and its duration since diagnosis. The secondary objective was to compare the academic delay possibly experienced by siblings with the general population of the same generation.

## 2 METHODS

### 2.1 Study design and population

#### 2.1.1 LEA program and population

The Leucémies de l'Enfant et de l'Adolescent (LEA) project is a French, multicenter, prospective program aiming to follow‐up in the long term all childhood AL survivors treated before the age of 18 years in the participating centers since 1980. Patient inclusion starts 1 year after finishing treatment, and evaluations performed through standardized medical consultations are repeated every 2 or 4 years depending on current age and years from diagnosis or last relapse. The LEA program, approved by a research ethics committee (Committee for Personal Protection), has been previously described,^25^ and details are available at www.plateforme‐lea.fr.

In 2017, we conducted an ancillary study called FRA‐LEA to assess the perceived QoL among survivors' siblings, long after AL diagnosis.^15^ For this original study, families of patients enrolled in one of the centers participating in the LEA program were contacted by letter. Data from eligible siblings who were at least 8 years of age at assessment and who had the narrowest age gap with the survivor were collected.

For our present study, we included siblings issued from FRA‐LEA when their date of birth matched with the general population's generation, but those who did not complete the part of questionnaire focusing on the educational outcomes and half‐siblings who were not living together at diagnosis, were not included.

At the time of assessment in 2017, school was compulsory in France from ages 6 to 16 (from age 3 since 2019). Middle school is for 4 years, with the official age of entry into grade called “sixième” being 11 years. The age indicators “on time” and “late” are calculated according to the theoretical reference age‐for‐grade, that is, 11 years old in “sixième,” 12 years old in “cinquième,” 13 years old in “quatrième,” and 14 years old in “troisième.” However, due to grade repetitions, students are getting overage for their grade and thus could complete middle school by the age of 16. This suggests that students who enter 2 years late, aged 16 at the start of the 1993 school year, were born in 1977. Similarly, students who enter on time, aged 11 in the 2014 school year, were born in 2003. Basically, the generation evaluated in our study included individuals who were born between the years 1977 and 2003.

#### 2.1.2 Comparison group—French population

Educational data from the French general population are from the Direction de l'évaluation, de la performance et de la prospective (DEPP) of the national education ministry. These data concern French students enrolled in middle school, from 1993 to 2014.^26^

The statistics from the DEPP present the number and the rate of all students who are “on‐time” or “late” in each of the four middle school grades (“sixième” to “troisième”) and for each year, from 1993 to 2014.

### 2.2 Collected data

All information was collected from the FRA‐LEA study or the LEA program and was available for this study.

#### 2.2.1 About siblings

‐ Sociodemographic data: sex, age at evaluation, month of birth (categorized as the first part of the year from January to June and the second half of the year from July to December), type of sibling relationship (twin, brother or sister, half‐brother or sister), and birth order.
‐ Health‐related data: donor or not if the survivor received an allogeneic hematopoietic stem cell transplantation (HSCT), and health status, implying the presence/absence of chronic disease.
‐ Education‐related information includes school difficulties, defined as the occurrence of a grade repetition; if yes, the time according to the diagnosis (before, during, or after) and the grade, absenteeism due to the disease, orientation choice (professional/vocational or not), current education status; if out of school, the level attained, the employment status (currently working or not) and the profession.

#### 2.2.2 About the family

Data about the family environment at the time of diagnosis:

‐ The socioeconomic situation, indicating household financial difficulties, was assessed using a 5‐point Likert scale with answer choices ranging from “very at ease” to “very difficult.”
‐ The parent's education, reflecting the highest level of education attained, was evaluated in two categories: high school graduate or not.
‐ The parent's employment status was provided, and their availability during treatment was considered when at least one of them stopped working, either temporarily or permanently.

#### 2.2.3 About the survivor

‐ Sociodemographic data: sex, age at diagnosis and the occurrence of repeating a grade.
‐ Cancer‐related information and treatments were detailed: period of diagnosis, time since diagnosis, subtype of AL, occurrence of relapse, use of central nervous system (CNS) irradiation, total body irradiation (TBI), and HSCT. An overall burden index was computed as follows: relapse (1 point), irradiation (CNS or TBI, 1 point), and HSCT (1 point), resulting in a 3‐point rating scale.^13^
‐ The number of late physical effects, consistently reported during each specific medical visit, was also documented.

### 2.3 Statistical analyses

Categorical variables are presented as numbers and percentages, while quantitative data are reported using mean ± standard deviation (*SD*).

Within the eligible siblings, we assessed the representativeness of our sample by comparing survivors' sociodemographic and cancer‐related characteristics between siblings' participants and nonparticipants using chi‐squared test and Student's *t*‐test (for percentages and means, respectively).

To explore factors associated with postdiagnosis grade repetition, a binary logistic regression was used with the dependent variable coded as siblings who did not repeat a grade (never occurred or happened only before the AL diagnosis) versus those who repeated a grade after the AL diagnosis. All characteristics of the siblings, the family, and the survivor were compared.

Then, to better understand the link of those same factors with the time between the diagnosis and the siblings' grade repetition, a multinomial logistic regression was performed with three groups of siblings, as follows: those who did not repeat a grade (Group 1), those who repeated at short‐ or medium‐term, meaning in 5 years postdiagnosis (Group 2), and those who repeated at long‐term, meaning more than 5 years postdiagnosis (Group 3). Post hoc analysis was carried out to identify differences between Groups 2 and 3 in comparison with Group 1.

For both regressions, relevant variables were selected based on a *p*‐value ≤0.10 in the univariate analysis. To provide the best fit for the models, care was taken to remove relatively correlated or redundant independent variables. Additionally, the sex of the sibling and the time between the evaluation and the diagnosis were included in a systematic way as adjustment variables. Adjusted odds ratios (OR) and 95% confidence intervals were estimated.

For the comparison of our sample with the French general population, aiming to evaluate if siblings are more at risk of being late in their school career after the diagnosis, the distributions of students entitled “on time” and “late” expected in our cohort were established based on the distributions in France. Comparisons between the observed and expected distributions in siblings were performed using chi‐squared or Fisher's exact tests, as appropriate.

All tests were two‐sided, and *p*‐value threshold for statistical significance was set to 5% (0.05). Statistical analyses were performed using IBM SPSS Statistics version 20.0 (SPSS, Inc., Chicago, IL, USA).

## 3 RESULTS

### 3.1 Participants

The 2106 families enrolled in the LEA program in 2017 were contacted, and of the 1936 eligible siblings for the original FRA‐LEA study, a total of 772 consented to participate.

The results of the comparison of participants' and nonparticipants' siblings, based on cancer‐related factors, are provided in Table S1. The two groups did not differ in survivor's sex, type of AL, history of relapse, transplantation with or without TBI, number of late physical effects, and overall burden index of AL. The nonparticipant group was less than one‐year older at diagnosis (*p* = 0.001) and had a more common use of CNS irradiation (*p* = 0.02), which is correlated with a more significant presence of older diagnoses in the nonparticipant group.

**FIGURE 1:**
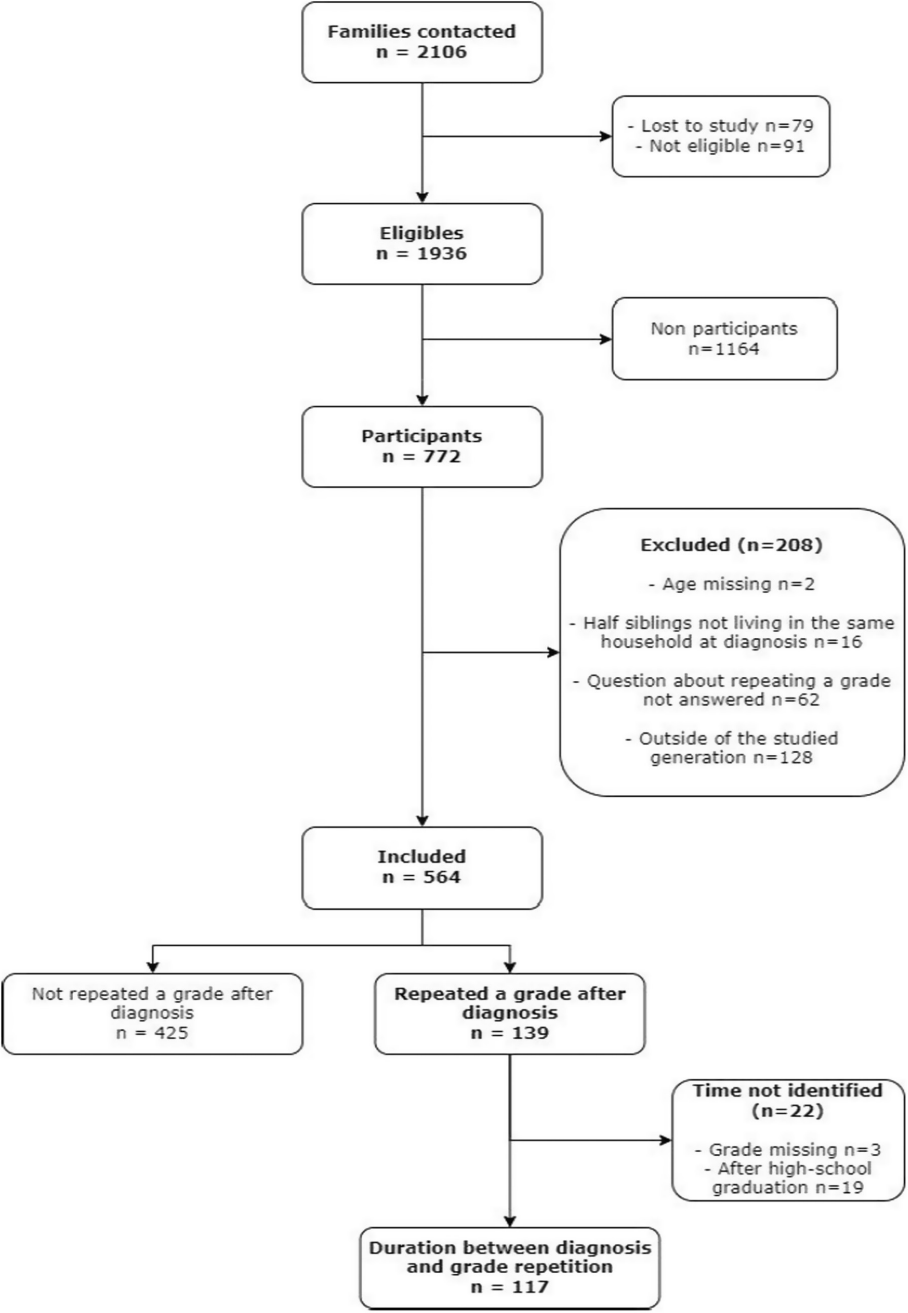
Flowchart.

Among the participants, 208 siblings met our exclusion criteria, of whom 128 were not part of the same generation of the French population, suggesting that they were born before 1977 or after 2003 and thus were never in middle school from 1993 to 2014. Ultimately, a total of 564 siblings were included in the analysis, as shown in the flowchart in Figure 1.

### 3.2 Siblings' descriptive characteristics

All descriptive characteristics of the 564 siblings included in our study are presented in Table 1.

**TABLE 1:** Siblings' characteristics.

	Total
	*n* = 564
*Sociodemographic and health‐related characteristics*	
Sex	
Male	239 (42.4)
Female	325 (57.6)
Age at evaluation (*μ* ± *σ*)	22.5 ± 6.8
<18 years	175 (31.0)
18–24 years	208 (36.9)
≥25 years	181 (32.1)
Age at survivor diagnosis (*μ* ± *σ*)	8.9 ± 5.1
Not yet born	30 (5.3)
<8 years	260 (46.1)
≥8 years	274 (48.6)
Years since survivor diagnosis	
(*μ* ± *σ*)	14.1 ± 6.4
(med [min; max])	12.8 [2.3; 35.5]
<10 years	160 (28.4)
≥10 years	404 (71.6)
Types of sibling	
Twins	13 (2.3)
Brothers and sisters	531 (94.3)
Half siblings	19 (3.4)
Birth order	
Younger	196 (34.8)
Older (or twins^a^)	368 (65.2)
Donor	
Yes	47 (8.3)
No	517 (91.7)
Chronic diseases^b^	
Yes	71 (12.7)
No	488 (87.3)
*Family characteristics*	
Financial situation^c^	
At ease or medium	375 (89.1)
Difficult	46 (10.9)
Parental educational level^d^	
At least one of them did not graduate	236 (58.4)
Both graduated from high school or higher	168 (41.6)
Parents' employment^e^	
None of them stopped working	93 (24.2)
At least one of them stopped working to take care of the ill child	292 (75.8)
*Academic and professional characteristics*	
Repeated a grade after the diagnostic	
No	425 (75.4)
Yes	139 (24.6)
If yes, how many times?	
Once	123 (88.5)
Twice	14 (10.1)
>2	2 (1.4)
If yes, when?^f^	
At least once before the end of high school	118 (86.1)
Only after high school	19 (13.9)
If before/during high school	
Age when repeated grade (*μ* ± *σ*)	14.4 ± 2.8
Time between diagnostic and grade repetition (*μ* ± *σ*)	6.4 ± 4.5
Time between diagnostic and grade repetition	
Short term (<2 years)	28 (23.9)
Medium term (2–5 years)	25 (21.4)
Long term (>5 years)	64 (54.7)
Repeated a grade before the diagnosis	
No	524 (92.9)
Yes	40 (7.1)
If yes, how many times?	
Once	37 (92.5)
Twice	3 (7.5)
>2	‐
Missing or temporary interrupting school (≥ 1 week)^g^	
No	531 (96.0)
Yes	22 (4.0)
If yes, how long? (*μ* ± *σ*)	2.7 ± 2.3
Professional/vocational education^h^	
No	398 (74.5)
Yes	136 (25.5)
End of education^i^	
No	307 (54.8)
Yes	253 (45.2)
If yes, which level?	
High school or lower	100 (39.5)
Higher education	153 (60.5)
If yes, currently working?	
No	31 (12.4)
Yes	220 (87.6)
If yes, which sector?	
Primary (agricultural activities)	6 (2.8)
Secondary (manufacturing/construction industries)	35 (16.6)
Tertiary (services)	170 (80.6)
*Including human health and social work activities*	40 (19.0)

*Note*: Missing data from “^b–i^”: *n* = 5, 143, 160, 179, 2, 11, 30, 4. μ: mean, σ: standard deviation.

^a^ *n* = 15 twins.

#### 3.2.1 Sociodemographic, health‐related and family characteristics

Most siblings were women (57.6%), with a mean age at evaluation of 22.5 ± 6.8 years. The overall mean time since diagnosis was 14.1 ± 6.4 years, and most siblings were older than the survivor (65.2%). Finally, more than 87% of the siblings did not report any chronic diseases.

The results of the siblings' family environment showed that 10.9% were from a family who experienced financial difficulties at the time of diagnosis, and in more than three‐fourth of the families, at least one of the parents stopped working during the treatment period.

#### 3.2.2 Academic and professional characteristics

Postdiagnosis grade repetition occurred for 139 (24.6%) of the siblings, and among the 118 siblings who repeated before graduating high school, the average duration from diagnosis to grade repetition was 6.4 ± 4.5 years, with a mean age at repetition of 14.4 ± 2.8 years. The description further indicated that 7.1% of them had a history of repeating a grade before the AL diagnosis. Of the 252 siblings who completed their education, 60.5% graduated from high school or higher, 87.6% were working at the time of assessment, and 40 of those active siblings had a health‐related or medico‐social profession.

### 3.3 Factors associated with postdiagnosis grade repetition in siblings: Bivariate logistic regression

Univariate and multivariate logistic regression analyses of predictors of postdiagnosis grade repetition were conducted and are presented in Table 2.

**TABLE 2:** Univariate and multivariate logistic regression.

	Not repeated a grade after the diagnosis	Repeated a grade after the diagnosis	OR (95% CI)	*p* Values	Adjusted OR (95% CI)	*p* Values
	(*n* = 425)	(*n* = 139)			*n* = 384	
Sibling's characteristics						
Sex				*0.23*		*0.21*
Male	*174 (40.9)*	*65 (46.8)*	*1.27 (0.86–1.86)*		*1.41 (0.83–2.40)*	
Female	*251 (59.1)*	*74 (53.2)*	*1*		*1*	
Age at evaluation (*μ* ± *σ*)	*21.5 ± 6.6*	*25.6 ± 6.5*	*1.09 (1.06–1.12)*	***<0.001***		
Age at evaluation				***<0.001***		
<18 years	*164 (38.6)*	*11 (7.9)*	*1*			
18–24 years	*144 (33.9)*	*64 (46.0)*	*6.63 (3.37–13.05)*			
≥25 years	*117 (27.5)*	*64 (46.0)*	*8.16 (4.12–16.13)*			
Years since survivor diagnosis (*μ* ± *σ*)	*13.3 ± 5.9*	*16.8 ± 6.9*	*1.09 (1.06–1.12)*	***<0.001***	*1.11 (1.06–1.53)*	***<0.001***
Years since survivor diagnosis				***0.001***		
<10 years	*136 (32.0)*	*24 (17.3)*	*1*			
≥10 years	*289 (68.0)*	*115 (82.7)*	*2.26 (1.39–3.66)*			
Months of birth				**0.02**		0.06
January–June	231 (54.4)	60 (43.2)	1		1	
July–December	194 (45.6)	79 (56.8)	**1.57 (1.07–2.31)**		1.64 (0.97–2.77)	
Age at survivor diagnosis				0.11		
Not yet born	26 (6.1)	4 (2.9)	1			
<8 years	202 (47.5)	58 (41.7)	1.87 (0.63–5.56)			
≥8 years	197 (46.4)	77 (55.4)	2.54 (0.86–7.52)			
Birth order				0.06		**0.006**
Younger	157 (36.9)	39 (28.1)	1		1	
Older (or twins)	268 (63.1)	100 (71.9)	1.50 (0.99–2.28)		2.34 (1.28–4.29)	
Donor				**0.01**		0.50
Yes	28 (6.6)	19 (13.7)	2.25 (1.21–4.16)		1.37 (0.55–3.43)	
No	397 (93.4)	120 (86.3)	1		1	
Chronic diseases^a^				0.12		
Yes	48 (11.4)	23 (16.5)	1.54 (0.90–2.63)			
No	372 (88.6)	116 (83.5)	1			
Repeated a grade before the diagnosis				0.74		
No	394 (92.7)	130 (93.5)	1			
Yes	31 (7.3)	9 (6.5)	0.88 (0.41–1.90)			
Family's characteristics						
Financial situation^b^				**0.01**		**0.008**
At ease or medium	291 (91.5)	84 (81.6)	1		1	
Difficult	27 (8.5)	19 (18.4)	2.44 (1.29–4.60)		2.81 (1.31–6.05)	
Parental educational level^c^				**0.002**		0.16
At least one of them did not graduate	168 (54.2)	68 (72.3)	2.21 (1.34–3.66)		1.51 (0.85–2.69)	
Both graduated from high school or higher	142 (45.8)	26 (27.7)	1		1	
Mother employability^d^				0.98		
Yes	250 (77.4)	76 (77.6)	1			
No	73 (22.6)	22 (22.4)	0.99 (0.58–1.70)			
Father employability^e^				0.71		
Yes	308 (97.5)	89 (96.7)	1			
No	8 (2.5)	3 (3.3)	1.30 (0.34–4.99)			
Parents' employment^f^				0.78		
None of them stopped working	70 (23.8)	23 (25.3)	1.08 (0.63–1.86)			
At least one of them stopped working to take care of the ill child	224 (76.2)	68 (74.7)	1			
Survivor's characteristics						
Age at diagnosis (*μ* ± *σ*)	6.5 ± 4.4	6.1 ± 4.2	0.98 (0.94–1.02)	0.35		
Months of diagnosis				0.96		
January–March	100 (23.5)	30 (21.6)	1			
April–June	113 (26.6)	38 (27.3)	1.12 (0.65–1.94)			
July–September	114 (26.8)	37 (26.6)	1.08 (0.62–1.88)			
October–December	98 (23.1)	34 (24.5)	1.16 (0.66–2.03)			
Sex				0.99		
Male	205 (48.2)	67 (48.2)	1			
Female	220 (51.8)	72 (51.8)	1.00 (0.68–1.47)			
Type of acute leukemia				0.36		
Lymphoblastic	368 (86.6)	116 (83.5)	1			
Myeloblastic	57 (13.4)	23 (16.5)	1.28 (0.76–2.17)			
Relapse				***0.02***		
Yes	*40 (9.4)*	*23 (16.5)*	*1.91 (1.10–3.32)*			
No	*385 (90.6)*	*116 (83.5)*	*1*			
HSCT				***0.03***		
No	*347 (81.6)*	*101 (72.7)*	*1*			
Yes without TBI	*29 (6.8)*	*19 (13.7)*	***2.25 (1.21–4.18)***			
Yes with TBI	*49 (11.5)*	*19 (13.74)*	*1.33 (0.75–2.37)*			
CNS irradiation				*0.96*		
Yes	*30 (7.1)*	*10 (7.2)*	*1.02 (0.49–2.15)*			
No	*395 (92.9)*	*129 (92.8)*	*1*			
Burden index of leukemia				**0.03**		0.99
0–2	398 (93.6)	122 (87.8)	1		1	
3	27 (6.4)	17 (12.2)	2.05 (1.08–3.90)		1.01 (0.40–2.56)	
Repeated a grade^g^				**<0.001**		**0.001**
No	265 (73.0)	57 (49.1)	1		1	
Yes	98 (27.0)	59 (50.9)	2.80 (1.82–4.31)		2.48 (1.43–4.30)	
*p* Value Hosmer‐Lemeshow						*0.759*

*Note*: Missing data from “^a–g^”: *n* = 5, 143, 160, 143, 156, 179, 85. Italic values: adjustment or redundant variables. Bold values: *p*‐value <0.05 was significant. μ: mean, σ: standard deviation.

Abbreviations: CNS, central nervous system; HSCT, hematopoietic stem cell transplantation; TBI, total body irradiation.

The results of the univariate analysis indicated that, compared with the non‐repeater siblings, those who repeated a grade after the AL diagnosis were more likely to be older than the survivor (*p* = 0.06), born in the second half of the year (*p* = 0.02), and donors of HSCT (*p* = 0.01). Parents of siblings who did not repeat postdiagnosis were more likely to both have graduated from high school or higher (*p* = 0.002) and faced fewer financial difficulties at diagnosis (*p* = 0.01). In survivors' characteristics, history of repeating a grade (*p* < 0.001) and a maximum in the burden index of AL (*p* = 0.03) were linked to postdiagnosis grade repetition in siblings.

In the multivariate model, the siblings' age at evaluation, the survivor's relapse, and CNS irradiation status were not entered because of a strong interaction with the time since diagnosis and the burden index of AL, respectively.

After adjusting for siblings' sex and time since the diagnosis, the independent factors associated with siblings' repeating a grade after the diagnosis were the birth order (higher risk for older siblings; OR 2.3 [1.28–4.29], *p* = 0.006), the household socioeconomic situation (higher risk if parents reported financial difficulties; OR 2.8 [1.31–6.05], *p* = 0.008), and the survivor's history of repeating a grade (higher risk if the survivor experienced it; OR 2.5 [1.43–4.30], *p* = 0.001).

### 3.4 Risk factors for repeating a grade in the short term versus long term: Multinomial regression

Data on the potential determinants, according to the time between the diagnosis and the siblings' grade repetition by multinomial logistic regression, are presented in Table 3.

**TABLE 3:** Multinomial logistic regression.

	Group 1	Group 2	Group 3	*p* Values	Groups comparison	
					*p* Values G1 vs. G2	*p* Values G1 vs. G3
	Not repeated a grade after the diagnosis	Short‐ medium‐term ≤5 years	Long‐term >5 years			
	(*n* = 425)	(*n* = 53)	(*n* = 64)			
Sibling's characteristics						
Sex				*0.37*		
Male	*174 (40.9)*	*21 (39.6)*	*32 (50.0)*			
Female	*251 (59.1)*	*32 (60.4)*	*32 (50.0)*			
Age at evaluation (*μ* ± *σ*)	*21.5 ± 6.6*	*26.8 ± 7.0*	*24.6 ± 6.1*	***<0.001***		
Age at evaluation				***<0.001***		
<18 years	*164 (38.6)*	*6 (11.3)*	*4 (6.2)*			
18–24 years	*144 (33.9)*	*17 (32.1)*	*36 (56.2)*			
≥25 years	*117 (27.5)*	*30 (56.6)*	*24 (37.5)*			
Years since survivor diagnosis (*μ* ± *σ*)	*13.3 ± 5.9*	*15.3 ± 6.6*	*18.7 ± 6.8*	***<0.001***		
Years since survivor diagnosis				***<0.001***		
<10 years	*136 (32.0)*	*12 (22.6)*	*5 (7.8)*			
≥10 years	*289 (68.0)*	*41 (77.4)*	*59 (92.2)*			
Months of birth				0.08	0.12	0.06
January–June	231 (54.4)	23 (43.4)	27 (42.2)			
July–December	194 (45.6)	30 (56.6)	37 (57.8)			
Age at survivor diagnosis				**<0.001**		
Not yet born	26 (6.1)	0	4 (6.2)		‐	0.29
<8 years	202 (47.5)	12 (22.6)	37 (57.8)		**<0.001**	0.89
≥8 years (referent)	197 (46.4)	41 (77.4)	23 (35.9)			
Birth order				**<0.001**	**<0.001**	0.54
Younger	157 (36.9)	4 (7.5)	28 (43.8)			
Older (or twins)	268 (63.1)	49 (92.5)	36 (56.2)			
Donor				**0.04**	0.42	**0.018**
Yes	28 (6.6)	5 (9.4)	10 (15.6)			
No	397 (93.4)	48 (90.6)	54 (84.4)			
Chronic diseases^a^				0.51	0.50	0.58
Yes	48 (11.4)	8 (15.1)	10 (15.6)			
No	372 (88.6)	45 (84.9)	54 (84.4)			
Repeated a grade before the diagnosis				0.05	0.07	0.25
No	394 (92.7)	46 (86.8)	63 (98.4)			
Yes	31 (7.3)	7 (13.2)	1 (1.6)			
Family's characteristics						
Financial situation^b^				**<0.001**	**<0.001**	0.24
At ease or medium	291 (91.5)	23 (65.7)	43 (86.0)			
Difficult	27 (8.5)	12 (34.3)	7 (14.0)			
Parental educational level^c^				**0.001**	**0.003**	0.33
At least one of them did not graduate	168 (54.2)	27 (87.1)	32 (69.6)			
Both graduated from high school or higher	142 (45.8)	4 (12.9)	14 (30.4)			
Mother employability^d^				0.83	0.64	0.95
Yes	250 (77.4)	27 (79.4)	34 (73.9)			
No	73 (22.6)	7 (20.6)	12 (26.1)			
Father employability^e^				0.54	‐	0.52
Yes	308 (97.5)	27 (100.0)	45 (95.7)			
No	8 (2.5)	0	2 (4.3)			
Parents' employment^f^				0.62	0.22	0.62
None of them sto pped working	70 (23.8)	5 (16.1)	11 (24.4)			
At least one of them stopped working to take care of the ill child	224 (76.2)	26 (83.9)	34 (75.6)			
Survivor's characteristics						
Age at diagnosis (*μ* ± *σ*)	6.5 ± 4.4	6.6 ± 4.3	4.7 ± 3.3	**0.006**	0.97	**0.002**
Months of diagnosis				0.39		
January–March	100 (23.5)	7 (13.2)	20 (31.2)		0.09	0.08
April–June	113 (26.6)	15 (28.3)	17 (26.6)		0.71	0.29
July‐September	114 (26.8)	15 (28.3)	16 (25.0)		0.71	0.32
October–December (referent)	98 (23.1)	16 (30.2)	11 (17.2)			
Sex				0.88	0.83	0.44
Male	205 (48.2)	26 (49.1)	33 (51.6)			
Female	220 (51.8)	27 (50.9)	31 (48.4)			
Type of acute leukemia				0.71	0.84	0.18
Lymphoblastic	368 (86.6)	46 (86.8)	53 (82.8)			
Myeloblastic	57 (13.4)	7 (13.2)	11 (17.2)			
Relapse				***0.024***		
Yes	*40 (9.4)*	*8 (15.1)*	*13 (20.3)*			
No	*385 (90.6)*	*45 (84.9)*	*51 (79.7)*			
HSCT				*0.13*		
No	*347 (81.6)*	*41 (77.4)*	*45 (70.3)*			
Yes without TBI	*29 (6.8)*	*4 (7.5)*	*10 (15.6)*			
Yes with TBI	*49 (11.5)*	*8 (15.1)*	*9 (14.1)*			
CNS irradiation				*0.80*		
Yes	*30 (7.1)*	*4 (7.5)*	*6 (9.4)*			
No	*395 (92.9)*	*49 (92.5)*	*58 (90.6)*			
Burden index of leukemia				0.31	0.14	0.37
0–2	398 (93.6)	46 (86.8)	55 (85.9)			
3	27 (6.4)	7 (13.2)	9 (14.1)			
Repeated a grade^g^				**<0.001**	0.14	**<0.001**
No	265 (73.0)	26 (59.1)	20 (37.0)			
Yes	98 (27.0)	18 (40.9)	34 (63.0)			

*Note*: Missing data from “^a–g^”: *n* = 5, 139, 155, 139, 152, 172, 81. Italic values: adjustment or redundant variables. Bold values: *p*‐value <0.05 was significant. μ: mean, σ: standard deviation.

Abbreviations: CNS, central nervous system; HSCT, hematopoietic stem cell transplantation; TBI, total body irradiation.

The results showed that factors significantly linked with grade repetition in the 5 years postdiagnosis were the sibling's age at diagnosis (*p* < 0.001), the birth order (*p* < 0.001), the household socioeconomic situation (*p* < 0.001), and parents' educational level (*p* = 0.003): an age of ≥8 years old, being older, parents reporting financial difficulties, and having one of them who did not graduate, put siblings at higher risk for repeating a grade in the short term after diagnosis.

Significant factors associated with a grade repetition more than 5 years postdiagnosis were the siblings' HSCT donation (*p* = 0.018), the survivor's age at diagnosis (*p* = 0.002), and their history of repeating a grade (*p* < 0.001). Being a donor of HSCT and having a survivor who was younger at diagnosis and who already repeated a grade, put siblings at higher risk for repeating a grade long after the diagnosis.

### 3.5 Comparison with the French general population

The approach used in our study consisted of focusing on the rate of grade repetition in the last grade of middle school. Compared with the French population, in the studied period from 1993 to 2014, the differentials between the observed and expected distributions were all negative. These results suggest that siblings were more “on time” at the end of middle school (Table 4).

**TABLE 4:** Comparison with the French general population—last grade of middle school.

	General population	Study population	Study population	Differential	*p* Values	Comments
	*n* (%)	Observed	Expected	O – E		
1993					1	In 1989, the “cycles reform” prevents a student from repeating a grade more than once in primary school. Consequence: from 1990, increase of grade repetition in the first year of middle school
On‐time (14 years old)	393,455 (50.5)	3	2.53			
Delayed (15 years old and more)	361,832 (46.5)	2	2.33	−0.33		
1997					0.40	Highest rate of grade repetition registered
On‐time	411,765 (49.3)	9	5.92			
Delayed	399,650 (47.8)	3	5.74	−2.74		
2000					0.11	Resistance to grade repetition practice. −4% from 1993 to 2000
On‐time	449,187 (54.8)	13	8.22			
Delayed	347,335 (42.4)	2	6.36	−4.36		
2003					0.23	France, first OECD country to make students repeat grades (PISA 2003)
On‐time	472,523 (57.3)	16	12.03			
Delayed	329,623 (40.0)	5	8.4	−3.40		
2006					**0.03**	Stable rates of grade repetition
On‐time	483,254 (59.9)	24	16.17			
Delayed	300,450 (37.2)	3	10.04	−7.04		
2009					**0.03**	Sharp decline. −6% from 2006 to 2009
On‐time	512,056 (65.5)	35	26.2			
Delayed	244,072 (31.2)	5	12.48	−7.48		
2012					0.93	From 2009 to 2012, France recorded the second best performance among OECD countries in decrease of grade repetition. (PISA 2012)
On‐time	566,415 (70.6)	18	17.65			
Delayed	208,812 (26.0)	7	6.5	0.50		
2014					0.14	Between 1993 and 2014: −25%
On‐time	627,081 (75.0)	26	21.00			
Delayed	182,942 (21.9)	2	6.13	−4.13		

*Note*: Bold values: *p*‐value <0.05 was significant.

Abbreviations: OECD, Organization for Economic Co‐operation and Development; PISA, Programme for International Student Assessment.

## 4 DISCUSSION

In our study, we reported that one‐fourth of siblings repeated a grade after diagnosis, which is higher than what has been previously reported by Bonneau et al. but still lower in comparison with AL survivors,^27^ suggesting that healthy siblings were less likely to be held back in school than survivors. To our knowledge, when comparing both the ill child and their siblings, there is no additional research on grade repetition specifically, but several studies using various educational outcomes, such as specialized education or high school graduation, conveyed the same reassuring message concerning siblings of childhood cancer survivors.^28^, ^29^, ^30^

Regarding the time of grade repetition according to the diagnosis, the most recent study of Bonneau et al.^24^ reported a mean of 4 years after diagnosis in survivors whereas our results showed that siblings repeated 6 years postdiagnosis on average, indicating that siblings could be more at risk for repeating a grade than survivors. As far as we know, no other study is available to compare our results with, but the shorter interval reported in the diagnosed child can be corroborated by several studies implying that the treatment side‐effects, such as TBI, resulting in cognitive disorders, but also absenteeism, can be associated with early disruptions in their schooling.^21^, ^31^, ^32^

In previous scientific literature, when siblings of childhood cancer survivors are compared to peers, several studies have reported difficulties concentrating, lower school attendance and thus, poorer school performance.^16^, ^18^, ^33^ These findings reported shortly after diagnosis and mostly by parents or teachers are concerning, but the comparison of our population of siblings of AL survivors to the French general population indicated that siblings who went through middle school from 1993 to 2014 did not face more academic delay during their school career. Our results are supportive and consistent with a review from Alderfer et al.,^5^ showing that siblings of childhood cancer survivors are similar to norms regarding grade retention.

Given that the mean age at grade repetition in siblings is 14 years old in our study, this result signifies that siblings are more vulnerable to repeating a grade at the end of middle school according to the French education standards' age‐for‐grade. This is consistent with a report from the French school ministry from 2014, which stated that “troisième” was the most repeated grade.^34^ This last year of middle school is also the time for students to decide between general or vocational/professional education. Regarding these crossroads, our study showed that 25.5% of our population of siblings took the latter path, which is very similar to the rate of 23.3% reported by the DEPP.^35^

In the subset of adults' siblings who completed their education and were working at the time of assessment, we specifically investigated their job sectors and occupations, as referenced by the National Institute of Statistics and Economic Studies (INSEE). While the distribution in sectors is coherent with the French data, the rate of siblings employed in human health and social work activities is higher (19% vs. 14.6%).^36^ This distinctive feature could be related to the positive impact of the disease commonly reported in the literature and affecting healthy brothers and sisters, such as a gain in empathy and compassion toward others, and a desire to help people.^5^

If mixed findings on siblings' schooling have been frequently reported, consideration of risk factors for grade repetition revealed that several characteristics were more consistent, resulting in valuable information for screening targets.

In siblings' characteristics, the first identified determinant for repeating a grade was their birth order, a factor usually reported in siblings' psychosocial difficulties.^7^, ^13^, ^37^ In our study, older siblings were even more at risk of repeating a grade in the 5 years after diagnosis. Regarding hematopoietic stem cell donors, previous studies have reported that they were more likely to experience emotional distress, such as post‐traumatic stress reactions, anxiety, and low self‐esteem.^38^, ^39^, ^40^ Our findings have also indicated that this process puts siblings at higher risk of repeating a grade in the long term and thus provides an impulse for further investigation in this subgroup. Last, because the start of the school year is in September in France, being born at the end of the year implies being the youngest in the classroom, but this characteristic was not found to be detrimental for siblings' educational achievement.

Other determinants associated with postdiagnosis repetition included family‐level factors, as more often than other brothers and sisters, siblings who repeated were from households with financial difficulties or lower school‐level, significantly increasing their probability of repeating a grade in the short term after diagnosis. Disadvantaged family socioeconomic status and parental education have previously been reported as risk factors for academic performance in children with cancer^24^, ^31^ and for child functioning in the general population.^41^, ^42^ Hence, our research adds evidence regarding the well‐known negative impact of social inequalities on health.

Among survivor's characteristics, two determinants were found to be linked with siblings' grade repetition after diagnosis: survivor's age at diagnosis and history of grade repetition. These results are consistent with the literature, as a younger age at diagnosis has commonly been identified as a significant risk factor for adverse cognitive problems,^43^, ^44^ resulting in a lower level of education in childhood leukemia survivors^45^ and thus, possibly affecting the sibling's schooling in return.

Overall, siblings with supposed weaknesses, whether medical‐ (presence of chronic diseases) or educational‐related (history of repeating a grade), were not more likely to report a negative outcome, just as the AL burden index did not impact the occurrence of grade repetition in siblings. Additionally, factors associated with short‐term repetition were sociodemographic data (birth order and socioeconomic status), while factors related to the disease (age at diagnosis and HSCT donor) were linked to long‐term repetition.

These observations underscore that beyond the severity of the cancer, the experience itself causes challenges, contributing to the general key message of our study.

### 4.1 Limitations and strengths

The main limitation of our present study that needs to be taken into consideration is our principal criterion for judgment. Indeed, although repeating a grade is a standard practice for underperforming students, it is both a collective and an institutional decision, dependent on national education policies. Hence, our criterion, which has been the subject of intense public debate, can be considered to vary over time, conforming to the political context. Moreover, grade repetition and its time of occurrence according to the diagnosis were assessed through a self‐report questionnaire, and information concerning any school difficulties prior to the disease was not collected. Thus, there may be a lack of precision in data collection, as we were unable to carry out a more detailed analysis of the school career.

Nevertheless, unlike some European countries, France does not practice automatic promotion, making grade repetition a relatively common and widespread practice, but also an impartial criterion that enables comparison with the general population. Finally, as previously stated by UNESCO, timely progression through school is an important indicator of progress and performance that can result from “academic failure, unsatisfactory progress, insufficient examination marks to advance to the next level of instruction, age, and poor attendance or simply from lack of local educational opportunities”.^46^ Another limitation concerns the population: as we only included siblings of survivors and those with the narrowest age gap, our results could possibly underestimate repercussions in other siblings of the family or compared to bereaved siblings.

The principal strength of our study is its valuable contribution to the literature involving siblings of childhood leukemia survivors. Indeed, by aiming to better understand the uniqueness of siblings' experience, our study addresses important, but understudied, aspects of long‐term repercussions in healthy brothers and sisters. Additionally, because siblings are the population of interest in our study and not the comparison group as usually reported in the literature, the importance of our results is reinforced. The second strong point is the LEA program, which, as a well‐designed cohort study, offers various advantages: (a) our sample size places our study as one of the largest published studies on sibling populations to date; (b) this multicenter cohort covers half of pediatric hematology‐oncology centers in France and thus, is highly representative at a national level; and (c) the quantity and quality of the data collected allowed us to explore a large number of determinants.

### 4.2 Conclusion

With this study, we not only give voice to healthy siblings' concerns but also provide useful information that may be useful for understanding their situation. Indeed, although our findings are comforting, childhood cancer within the family can cause long‐term disruptions in siblings' school life, thus preventing them from reaching their full potential. In addition, while parents must keep paying attention to siblings' needs and possible challenges encountered in their academic performance, health care professionals should make sure that appropriate resources and effective support are available.

Additionally, with the identification of risk factors, our ability to screen for vulnerable brothers and sisters emphasized the necessity to develop specified or family‐centered programs to help parents and siblings with more acute needs to better cope and reduce long‐term repercussions.

Finally, strategies to optimize practice in the health care system and establish partnerships across ministries of health and education should be considered new public health policies.

## AUTHOR CONTRIBUTIONS

**Cindy Faust:** Formal analysis (lead); funding acquisition (equal); methodology (equal); writing – original draft (lead); writing – review and editing (lead). **Pascal Auquier:** Conceptualization (equal); funding acquisition (equal); supervision (equal); writing – review and editing (equal). **Virginie Gandemer:** Funding acquisition (equal); investigation (equal); resources (equal); supervision (equal); writing – review and editing (equal). **Yves Bertrand:** Funding acquisition (equal); investigation (equal); resources (equal); writing – review and editing (equal). **Marie‐Dominique Tabone:** Funding acquisition (equal); investigation (equal); resources (equal); writing – review and editing (equal). **Sophie Ansoborlo:** Funding acquisition (equal); investigation (equal); resources (equal); writing – review and editing (equal). **André Baruchel:** Funding acquisition (equal); investigation (equal); resources (equal); writing – review and editing (equal). **Jacinthe Bonneau:** Funding acquisition (equal); investigation (equal); resources (equal); supervision (equal); writing – review and editing (equal). **Jean‐Hugues Dalle:** Funding acquisition (equal); investigation (equal); resources (equal); writing – review and editing (equal). **Pascal Chastagner:** Funding acquisition (equal); investigation (equal); resources (equal); writing – review and editing (equal). **Justyna Kanold:** Funding acquisition (equal); investigation (equal); resources (equal); writing – review and editing (equal). **Maryline Poirée:** Funding acquisition (equal); investigation (equal); resources (equal); writing – review and editing (equal). **Alexandre Theron:** Funding acquisition (equal); investigation (equal); resources (equal); writing – review and editing (equal). **Laura Olivier:** Funding acquisition (equal); investigation (equal); resources (equal); writing – review and editing (equal). **Isabelle Pellier:** Funding acquisition (equal); investigation (equal); resources (equal); writing – review and editing (equal). **Gérard Michel:** Funding acquisition (equal); investigation (equal); resources (equal); supervision (equal); writing – review and editing (equal). **Julie Berbis:** Investigation (equal); resources (equal); supervision (equal); writing – review and editing (equal).

## FUNDING INFORMATION

Supported by grants from the French National Clinical Research Program (PHRC‐N), the French National Cancer Institute (INCa) (PHRC‐K), and the GIRCI Méditerranée (AAP_VALODATA_2022).

## CONFLICT OF INTEREST STATEMENT

The authors declare no conflict of interest.

## ETHICS STATEMENT

This study was approved by the SUD MÉDITERRANÉE Ethics Committee (reference RO – 2016/20).

## CONSENT STATEMENT

This is an observational study. All participants received an information note and had the right to withdraw consent at any time.

## Supporting information


Table S1.

Appendix S1.

## Data Availability

The datasets analyzed during the current study are available from the corresponding author on reasonable request.
