# Supplementary material for: Educational outcomes in siblings of childhood leukemia survivors: Factors associated with school difficulties and comparison with general population
Source: Cancer Med. 2024 Jan 10;13(3):e6821. doi: 10.1002/cam4.6821 (PMC10904966; doi:10.1002/cam4.6821)
Supplement: Supplementary file 2 — Appendix S1. [file CAM4-13-e6821-s001.docx]

**Members of the L.E.A. study group**

**Marseille**: AUQUIER Pascal, BARLOGIS Vincent, BERBIS Julie, BERGEROT Astrid, CHAMBOST Hervé, CURTILLET Catherine, HAMIDOU Zeinab, MICHEL Gérard, ROMANO David, ROUSSEL Aphaia, SAULTIER Paul, SHAWKET Alaa, STERIN Arthur, SZEPETOWSKI Sarah, VISENTIN Sandrine, THURET Isabelle

**Nancy:** CHASTAGNER Pascal, DETRAIT M, MANSUY Ludovic, MERLIN Marie-Sophie, NUI Laurence, PERROT Aurore, PHULPIN Aurélie, POCHON Cécile, ROTH-GUEPIN Gabrielle, RUBIO M-Thérèse, DEUTSCH Hélène, KEMPF Antoine

**Nice**: BENADIBA Joy, DUHIL de BENAZE Gwénaelle, CHAMPENOIS Isabelle, MEYRONET Marie POIREE Maryline, PONDROM Morgane, ROHRLICH Pierre-Simon, SOLER Christine

**Clermont-Ferrand**: DORE Eric, GIRARD-MONIN Pauline, GREZE Victoria, ISFAN Florentina, KANOLD Justyna, MERLIN Etienne, ROUEL Nadège,

**Grenoble**: ADJAOUD Dalila, ARMARI-ALLA Corinne, BOBILLOT-CHAUMONT Séverine, MARTIN M-Pierre, PAGNIER Anne, PERRET Cécile, PLANTAZ Dominique

**Lyon**: BERTRAND Yves, CREAULO Anthony, GARNIER Nathalie, GAUTHIER Alexandra, HALFON-DOMENECH Carine, HU Julie-Yi, KEBAÏLI Kamila, OUACHE-CHARDIN Marie, RENARD Cécile

**Paris Trousseau**: ALIMI Aurélia, AUVRIGNON Anne, BOUAYAD AGHA Latéfa, BOUTROUX Hélène, DOLLFUS Catherine, DONADIEU Jean, GOURAUD Françoise, HERITIER Sébastien, LANDMAN-PARKER Judith, LEVERGER Guy, PELLEGRINO Béatrice, PETIT Arnaud, TABONE Marie Dominique

**St Etienne**: BERGER Claire, DAVID Audrey, SOLER Catherine, STEPHAN Jean Louis, THOUVENIN-DOULET Sandrine

**Rennes**: BONNEAU Jacinthe, COUSIN Elie, GANDEMER Virginie, LAMOUR Anne- Marie, PERTUISEL Sophie, PROBERT Jamie, PUISEUX Chloé, TAQUE Sophie,

**Montpellier**: AKBARALY Tasnim, HAOUY Stéphanie, NORGIEUX Corentin, SAUMET Laure, SIRVENT Nicolas, SIRVENT Anne, THERON Alexandre, TEYSSIER Anne-Charlotte

**Paris St Louis – Robert Debré**: BARUCHEL André, BRETHON Benoît, DOURTHE Marie-Emilie, LEBLANC Thierry, MOUKOKO Marie-Noelle, RAY-LUNVEN Anne-France, AZARNOUSH Saba, DALLE Jean- Hugues, FAHD Raymonda, GRAIN Audrey, LE MOUEL Lou, YAKOUBEN Karima, STRULLU Marion

**Bordeaux**: ALADJIDI Nathalie, ANSOBORLO Sophie, DE BOUYN-ICHER Céline, DHUNPUNTH Chloé, DUCASSOU Stéphane, GUILLOT Laurène, JUBERT Charlotte, MERCHED Maria, NOTZ-CARRERE Anne, VERITE-GOULARD Cécile,

**Strasbourg:** BERGTHOLD Guillaume, PAILLARD Catherine, VITET Ludivine, SALMON Alexandra, SPIEGEL Alexandra

**Angers:** BRASME Jean-François, CONTANT Pierre, DE CARLI Emilie, DEMOOR Charlotte, DUPLAN Mylène, FAUCHERE Magali, PELLIER Isabelle, PROUST-HOUDEMONT Stéphanie, VEILLON Pascal

**Toulouse :** BERTOZZI-SALAMON Anne-Isabelle, BOULANGER Cécile, CASTEX M-Pierre, GAMBART Marion, PASQUET Marlène, PLAT Geneviève, SALEL Yu Jin

**StDenis :** BOHRER Sandrine, CADET Aurélie, CHAMBON Fanny, JEHANNE Mathilde, REGUERRE Yves,
